# Web-Based Group Conversational Intervention on Cognitive Function and Comprehensive Functional Status Among Japanese Older Adults: Protocol for a 6-Month Randomized Controlled Trial

**DOI:** 10.2196/56608

**Published:** 2024-07-11

**Authors:** Kumi Watanabe Miura, Takashi Kudo, Mihoko Otake-Matsuura

**Affiliations:** 1 Center for Advanced Intelligence Project RIKEN Tokyo Japan; 2 Japan Society for the Promotion of Science Tokyo Japan; 3 Department of Psychiatry Graduate School of Medicine Osaka University Osaka Japan

**Keywords:** randomized controlled trial, web-based intervention, communication technology, cognitive health, neural blood markers, social isolation, well-being

## Abstract

**Background:**

Social communication is a key factor in maintaining cognitive function and contributes to well-being in later life.

**Objective:**

This study will examine the effects of “Photo-Integrated Conversation Moderated by Application version 2” (PICMOA-2), which is a web-based conversational intervention, on cognitive performance, frailty, and social and psychological indicators among community-dwelling older adults.

**Methods:**

This study is a randomized controlled trial with an open-label, 2-parallel group trial and 1:1 allocation design. Community dwellers aged 65 years and older were enrolled in the trial and divided into the intervention and control groups. The intervention group receives the PICMOA-2 program, a web-based group conversation, once every 2 weeks for 6 months. The primary outcome is verbal fluency, including phonemic and semantic fluency. The secondary outcomes are other neuropsychiatric batteries, including the Mini-Mental State Examination, Logical Memory (immediate and delay), verbal paired associates, and comprehensive functional status evaluated by questionnaires, including frailty, social status, and well-being. The effect of the intervention will be examined using a mixed linear model. As a secondary aim, we will test whether the intervention effects vary with the covariates at baseline to examine the effective target attributes.

**Results:**

Recruitment was completed in July 2023. A total of 66 participants were randomly allocated to intervention or control groups. As of January 1, 2024, the intervention is ongoing. Participants are expected to complete the intervention at the end of February 2024, and the postintervention evaluation will be conducted in March 2024.

**Conclusions:**

This protocol outlines the randomized controlled trial study design evaluating the effect of a 6-month intervention with PICMOA-2. This study will provide evidence on the effectiveness of social interventions on cognitive function and identify effective target images for remote social intervention.

**Trial Registration:**

UMIN Clinical Trials UMIN000050877; https://tinyurl.com/5eahsy66

**International Registered Report Identifier (IRRID):**

DERR1-10.2196/56608

## Introduction

Remote intervention, which allows interventions to be conducted remotely through communication technology with participants, may benefit health and convenience. Remote interventions enable people with low accessibility to interventions due to disability, illness, location, or pandemic to participate in interventions and provide equitable health service. In particular, there has been increased attention to internet-based social interventions that alleviate social isolation since the COVID-19 pandemic [1-3] because of increased social isolation reported [4].

Social isolation is a major public health concern and determinant of healthy longevity. According to Lancet Reports from the *Lancet* Commission on Dementia, social isolation has been identified as one of the single potentially modifiable risk factors for dementia [5]. A growing body of literature has reported a link between social interactions and the risk of dementia [6-9]. Recent longitudinal meta-analyses [10] have demonstrated the integrated risks of poor social interaction and greater loneliness with the occurrence of dementia. This meta-analysis suggests that the strength of this association is comparable to other well-established risk factors, such as low education, depression in later life, and poor physical activity.

Psychological, behavioral, and biological pathways may link social interactions to health. Social interaction may play a significant role in maintaining mental health [11,12] and could be an important contributing factor to dementia [13] by providing a sense of connection with others and buffering perceived stress. Social interactions may also have behavioral effects. Interacting with others may promote appropriate health behaviors [14,15] by providing access to health information and social support. Biological mechanisms may also be mediated by physiological processes involved in cardiovascular, immune, and neuroendocrine functions [16-18].

In addition, communication, the basis of social interaction, is an intellectual activity. Communication requires multiple cognitive processes. Furthermore, the multifaceted brain processes nonverbal information, such as the other person’s voice [19], facial expressions [20], and emotions [21], as well as a variety of verbal information during communication. Such cognitive stimulation through social and conversational activities can contribute to the maintenance of the cognitive abilities of an individual.

Based on the evidence, we hypothesized that social communication-based interventions would be effective in maintaining cognitive function. Consequently, a series of our previous randomized controlled trials (RCTs) examined the effect of structural face-to-face group conversation intervention and consistently observed some positive effects on verbal fluency [22,23]. Our explanatory investigation using multimodal magnetic resonance imaging identified the brain network, brain regions, and brain structure that could benefit from the intervention. Despite the significant limitation regarding the lack of preintervention data, we observed higher resting-state functional connectivity between the left inferior frontal gyrus as a seed region and the temporal pole and middle frontal gyrus, which may reflect the brain network involved in the intervention effects [24]. Our voxel-based morphometric analysis also identified the candidate brain regions involved in the intervention mechanism, including the lateral prefrontal cortex [25]. Further, analysis using diffusion tensor imaging metrics suggested that left frontal white matter structures were candidates for white matter microstructural changes effected by the intervention [26]. Building on these findings, we have inferred that our conversational intervention may stimulate language-related function in the prefrontal lobe, serving as a potential mechanism underlying the observed effects.

We recently focused on remote social intervention to address the challenges of the COVID-19 pandemic and clinical face-to-face interventions in which functional decline reduces accessibility to interventions. We developed a remote smartphone-based group conversation intervention program called “Photo-Integrated Conversation Moderated by Application” (PICMOA) and conducted a 12-week RCT to determine the effectiveness of the program [27]. The PICMOA trial showed a positive intervention effect on semantic fluency for those who were good at using smartphones before the intervention. On the other hand, potential issues for PICMOA intervention were also suggested, such as the difficulty of operating a small smartphone for older people, the psychological burden of using smartphones and apps for older adults, the possibility that the stimulation of visual social interaction is small due to the small smartphone screen, and the short intervention period.

To address these challenges of smartphone-based interventions for older adults in a past trial, we developed Photo-Integrated Conversation Moderated by Application version 2 (PICMOA-2), a remote conversational intervention for PC tablets. By adapting the intervention to a PC tablet, a larger screen is expected to improve operability for older people and increase cognitive stimulation through recognition of the speaker’s face.

The primary aim of this study is to evaluate the effect of a 6-month intervention with PICMOA-2 on cognitive function in community-dwelling older adults. In addition to investigating the intervention’s effects on cognitive function, this study also evaluates its impact on frailty and social and psychological indicators. We hypothesized that this intervention may have secondary effects, potentially contributing to the physical, mental, and social well-being of participants. These possible secondary effects could arise from the maintenance of cognitive function through our intervention or via other pathways such as stress buffering, behavioral changes, and physiological processes owing to the enhanced social interaction by the intervention. Furthermore, this study will address whether target attributes modify the intervention effects as a secondary objective, as it remains unclear whether web-based conversational intervention is effective for a broad population or only for a specific population.

## Methods

### Study Design

The PICMOA-2 trial is an open-label, 6-month RCT with 2 parallel groups, including 1 intervention group and 1 passive control group. The overall timeline of the procedure is shown as a SPIRIT flow in Table 1. The main and secondary outcomes will be assessed at baseline and after a 6-month intervention period.

**Table 1:** Overall timeline of the procedure of the PICMOA-2 trial.

Time point				Enrollment		Allocation		Post allocation			Evaluation
				–t_1_		0		t_1_	t_2_		t_3_
**Enrollment**											
	Eligibility screening		✓		—^a^		—		—	—	
	Informed consent		✓		—		—		—	—	
	Allocation		—		✓		—		—	—	
**Interventions**											
	Practice using equipment and test session		—		—		✓		—	—	
	Interventions		—		—		—		✓	—	
**Assessments**											
	**List of baseline variables**										
		Demographic and socioeconomic information	✓		—		—		—	—	
		Lubben Social Network Scale-6	✓		—		—		—	—	
		Familiarity with using devices	✓		—		—		—	—	
	**List of outcome variables**										
		Mini-Mental State Examination	✓		—		—		—	✓	
		Verbal fluency tests	✓		—		—		—	✓	
		Logical Memory I and II	✓		—		—		—	✓	
		Verbal paired associates I and II	✓		—		—		—	✓	
		Kihon checklist	✓		—		—		—	✓	
		UCLA^b^ Loneliness Scale 10-item version	✓		—		—		—	✓	
		The 5-item World Health Organization Well-Being Index	✓		—		—		—	✓	
		Health Utility Index Mark 3	✓		—		—		—	✓	
		S-A creativity test	✓		—		—		—	✓	
	**List of other variables**										
		Blood-based biomarkers	—		—		✓		—	✓	
		Face scale	—		—		—		✓	—	

^a^Not applicable.

^b^UCLA: University of California, Los Angeles**.**

### Participants

**Figure 1:**
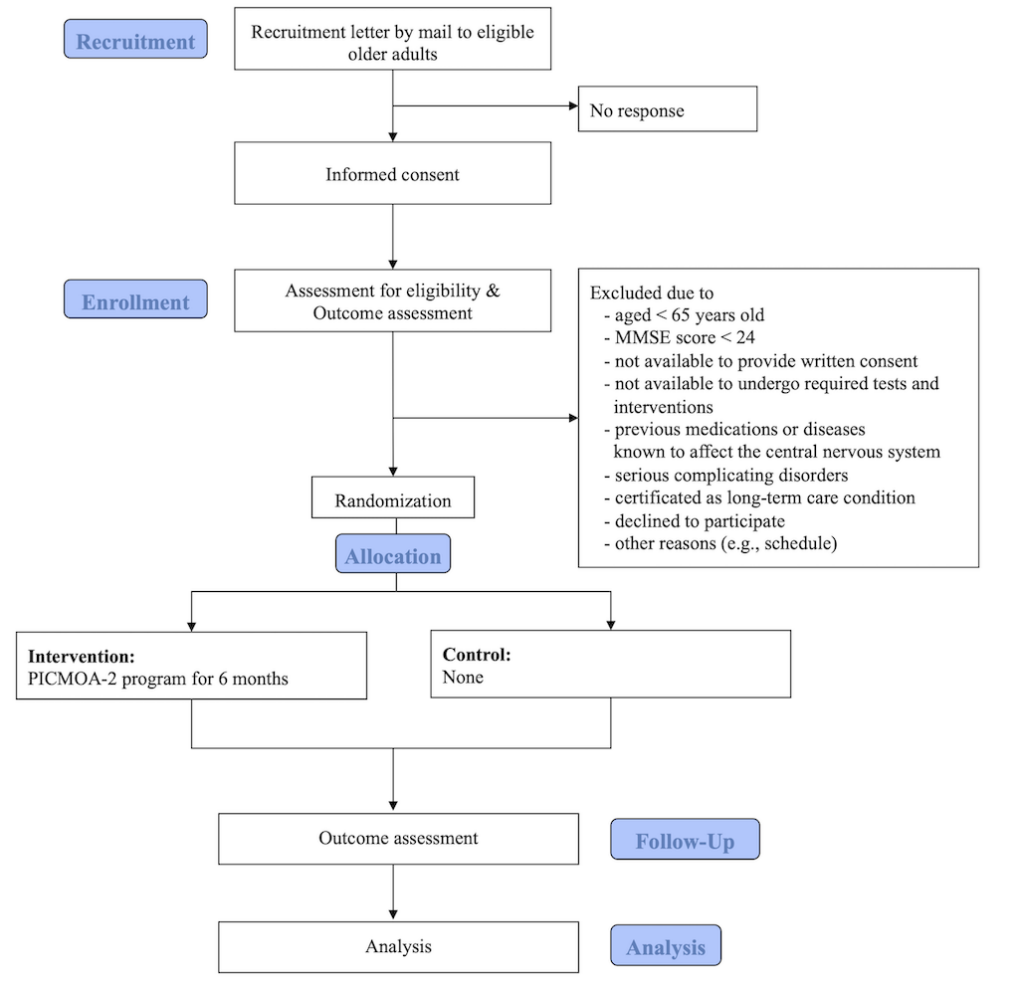
Participant flow of the PICMOA-2 trial. MMSE: Mini-Mental State Examination; PICMOA-2: Photo-Integrated Conversation Moderated by Application version 2.

We recruited community-dwelling older adults aged 65 years and older without cognitive decline living in Kishiwada city, Osaka Prefecture, Japan, near a metropolitan area with a population of 188,129 as of December 2023 [28]. This study’s design is illustrated in Figure 1. This procedure included eligibility screening, informed consent, outcome assessments, randomization, and 6 months of web-based conversational intervention. The inclusion criteria are as follows: a Mini-Mental State Examination (MMSE) score ≥ 24, available to provide written consent, and able to undergo the required tests and interventions on specified dates. We screened medical conditions and medication use and excluded patients with dementia; those with a history of disease or previous medications that could affect the central nervous system, such as neurological impairment, serious complicating disorders, and a history of serious head injury; and those certified as having a long-term care condition (“care needs levels” or “support need levels”) by the national public long-term care insurance system in Japan.

Participants were recruited by sending recruitment letters and flyers to community-dwelling older adults from the municipal government and posting announcements for enrollment in a new community paper. We mailed 3000 recruitment letters to randomly selected older adults without certificates of long-term care in 4 living areas of Kishiwada city. Some flyers were also distributed to the public and in collaboration with organizations. Once potential participants expressed interest in participating in the trial, they were screened for eligibility. Participants in both groups will receive cash compensation.

### Intervention

**Figure 2:**
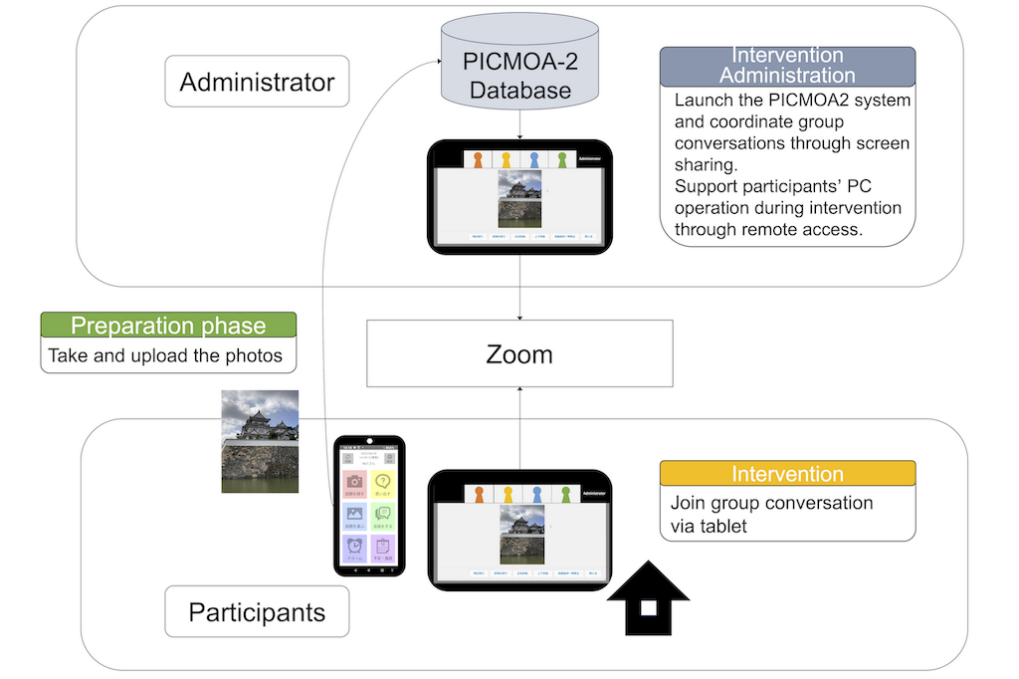
The intervention architecture. PICMOA-2: Photo-Integrated Conversation Moderated by Application version 2.

The intervention program is PICMOA-2, a web-based conversational intervention that uses a PC tablet at the participant’s home. Participants are instructed to take photos in their daily lives as much as possible according to the specific 12 themes during the 6-month intervention period and join conversational intervention based on PICMOA-2 once every 2 weeks for 6 months. Figure 2 describes the intervention structure of PICMOA-2. Participants are required to upload to the web system using a smartphone app that we originally developed before the intervention session, join the group conversational intervention session, and have a presentation and discussion about the photos that participants took during the session.

In the conversational session, each participant in a designated group of 4 gives a 1-minute speech about the 2 photos they took in their daily lives. Other participants then ask questions related to the photos, and the presenter answers the questions in the context of a natural conversation for 2 minutes for each photo. Research staff administer intervention using the PICMOA-2 system and support participants’ PC operations through remote access.

In the case of dropouts, substitutes are included in the group conversation to maintain the intensity of the intervention since different numbers of group members can lead to quantitative differences in the amount and duration of speaking, potentially impacting the effect sizes.

Smartphones for taking photos and PC tablets (Surface Go; Microsoft Corp) for conversational sessions with an internet connection were prepared for all participants.

### Outcomes

#### Overview

Table 2 shows the overall outcome measurements.

**Table 2:** The construction of outcome measurements.

Construction	Outcomes
Cognitive function	MMSE^a^, verbal fluency tests, Logical Memory I and II, and verbal paired associates I and II
Frailty	Kihon checklist
Loneliness	UCLA-LS 10^b^
Well-being	WHO-5^c^
Health-related quality of life	HUI3^d^
Divergent thinking	S-A creativity test

^a^MMSE: Mini-Mental State Examination.

^b^UCLA-LS 10: The University of California, Los Angeles Loneliness Scale-10 item version.

^c^WHO-5: 5-item World Health Organization Well-Being Index.

^d^HUI3: Health Utilities Index Mark 3.

#### Cognitive Functions

The main outcome measure is cognitive function. Specifically, we hypothesized that the PICMOA-2 may stimulate linguistic and executive functions through fundamental conversation activities based on the results of previous RCTs [22,23,27] and mechanistic hypothesis based on the explanatory investigation using multimodal magnetic resonance imaging [24-26]. Thus, we will specifically focus on verbal fluency as the primary outcome, which may reflect linguistic and executive functions. Well-trained clinical psychologists administered standardized neuropsychiatric tests to evaluate the effects of the intervention on cognitive function.

MMSE [29] is used to evaluate global cognitive function and screen for cognitive decline. The MMSE consists of 11 question sets and the total score is calculated. Verbal fluency tests, including 3 tasks each for semantic and phonemic fluency, are administered to assess linguistic and memory retrieval abilities, which may reflect executive control. A semantic fluency test is required to produce the names of animals, sports, and jobs, while the phonemic fluency test is required to generate the words that begin with the letter “a,” “ka,” and “si” in Japanese within 1 minute each. To evaluate episodic memory, Logical Memory I and II and the Verbal paired associates from the Wechsler Memory Scale-Revised [30] are introduced. Logical Memory is a story recall subtest. Logical Memory I assesses immediate recall and is required to immediately reproduce each of the 2 stories read out by the examiner, whereas Logical Memory II is required to reproduce stories approximately 30 (±5) minutes later to assess delayed recall. Verbally paired associates are paired-associate learning subtests. Participants are required to learn 4 pairs of related and unrelated words and test their memory of word pairs. Verbal Paired Association I assesses immediate recall, while Verbal Paired Association II assesses delayed recall approximately 30 (±5) minutes later.

#### Frailty

The Kihon checklist (KCL) [31] is used to assess frailty. The KCL is a self-report scale for frailty that has shown high validity in Japanese community-dwelling older adults. The KCL is commonly used in Japan, as municipalities across Japan have used it to determine high-risk populations to invite or evaluate intervention programs. The KCL includes 7 domains: daily life, physical strength, nutritional status, oral function, extent of house boundedness, cognitive function, and risk of depression. The total KCL scores and the occurrence and recovery of comprehensive or domain frailty will be assessed.

#### Social and Psychological Factors

Loneliness, well-being, health-related quality of life (HRQOL), and divergent thinking are introduced to evaluate the effects of conversational interventions on social and psychological aspects.

The University of California, Los Angeles Loneliness Scale-10 item version (UCLA-LS 10) [32] is used to assess loneliness. A total UCLA-LS score of 10 is calculated from the sum of each item (10-40 points). Higher scores on the UCLA-LS 10 indicate severe loneliness. The 5-item World Health Organization Well-Being Index [33] is introduced to evaluate well-being. The percentage score of WHO-5 is calculated (0-100 points) according to the manual. The Health Utilities Index Mark 3 [34] is used to assess HRQOL, and the multi-attribute utility score (–0.36 to 1.00) is calculated using the formula. Higher 5-item World Health Organization Well-Being Index and Health Utilities Index Mark 3 reflect better well-being and HRQOL. An item fluency test from the S-A creativity test [35], which is a divergent thinking assessment tool, is also introduced. While the S-A creativity test has 3 kinds of tasks that correspond to the 3 tasks of the Torrance test of creative thinking [36], including unusual use, product improvement, and just suppose, we only perform an unusual use task, which is an item fluency test to evaluate the flexibility of thought and divergent thinking, due to the balance between our study interest and time limitation. The 2 questions in the unusual use task ask participants to generate as many ideas as possible for the alternative uses of typical objects in unique ways in 5 minutes. The scoring of the S-A test is calculated using the formula by Tokyo Shinri Corporation.

#### Covariates

The covariates from the questionnaire include the following variables to describe participants’ demographics: age, sex, educational attainment, social isolation (the Lubben Social Network Scale-6) [37], and familiarity with devices such as smartphones, PCs, and email. Blood samples are also collected, with the measurement of blood-based biomarkers, including plasma neurofilament light chain (NfL), plasma tau, and tau in neuron-derived extracellular vesicles from plasma, which may reflect neuronal condition.

### Sample Size

We used G*power [38] to calculate the sample size, assuming the following conditions: 2-sided hypothesis test; medium effect size (f=0.25); 95% power; an analysis of variance model between groups over time; and 5% α level. Therefore, 65 participants are required, with a 20% dropout rate.

### Randomization and Blinding

Stratified block randomization, a block design (2, 4, and 6 block sizes) with a 1:1 allocation, was performed using the University Hospital Medical Information Network Internet Data and Information System for Clinical and Epidemiological Research Cloud version [39]. The participants were allocated to either the intervention or the control group. Sex (male or female) and MMSE scores (28 or higher and 27 or lower) at baseline were used for stratification. KWM is responsible for the randomization. As this study uses an open-label design, we aim to prevent potential assessment bias by blinding the assessors.

### Statistical Analysis

This study evaluates the effects of the PICMOA-2 intervention on outcome measurements. Linear mixed models with random intercepts for outcome measurements will be used to estimate the intervention effects. Our model will include the group assignment factor, time factor, and group × time interaction term and evaluate the intervention effect from the interaction term. As a secondary aim, we will test whether the intervention effects varied with the covariates at baseline to examine the effective target attributes.

All analyses will be conducted using the R software (The R Foundation). Statistical significance will be set at *P*<.05.

### Ethical Considerations

This trial was approved by the Ethics Committee of RIKEN (RIKEN-W1-2022-063) and was registered in the University Hospital Medical Information Network clinical trial registry (UMIN000050877).

## Results

Recruitment began in April 2023 and ended in July 2023. In total, 66 participants were enrolled and randomly allocated to intervention or control groups. As of January 1, 2024, the intervention is ongoing. Participants are expected to complete the intervention at the end of February 2024; the postintervention evaluation will be conducted in March 2024.

## Discussion

### Findings and Strength

This protocol aimed to evaluate the effect of a 6-month PICMOA-2 intervention, a web-based group conversational intervention, on cognitive function, frailty, and social and psychological indicators to determine the effectiveness of a web-based remote conversational intervention that we originally developed.

This study has several strengths. First, it may have clinical significance by accumulating evidence on the effectiveness of social interventions on cognitive function and digital health practices. Although large-scale observational studies have demonstrated a link between social interaction and cognitive health [2-7], whether social interventions can slow cognitive decline remains unknown. Since there is low evidence of social intervention and there are no clear clinical guidelines, this study may add knowledge on the effectiveness of social intervention. Second, this study examined whether there are differences in intervention effects depending on attributes by collecting subjective and laboratory data. A previous study in a completed clinical trial on mild Alzheimer disease suggested that baseline plasma NfL, which is a neurological biomarker, holds independent information on short-term cognitive decline [40]. Our previous RCT of a face-to-face conversational intervention program also found a positive intervention effect in the group with lower levels of NfL [23]. In addition, our previous PICMOA trial using smartphones suggested that familiarity with digital devices causes differences in the intervention effect [27]. This research may contribute to the search for clues to effective target images for remote social intervention by examining effective targets. Finally, this study directly recruited community-dwelling older adults mainly via mail in cooperation with the municipal government. This method can eliminate selection bias and enhance generalizability to real community intervention settings compared with recruiting from specific organizations or surveys.

### Limitations

This study uses an open-label trial design. Although the assessors are blinded to avoid assessment bias, blinding the research staff and participants is not possible for such behavioral interventions. In addition, the sample size was designed with a minimum of 65 participants for feasibility considerations. Large-scale validation is required to examine the effectiveness of the intervention.

### Conclusions

This protocol outlines the design of an RCT study that evaluates the effect of a 6-month intervention with PICMOA-2 on cognitive function, frailty, and social and psychological indicators. Though examining the effect of the PICMOA-2 program, this study will provide evidence of the effectiveness of social interventions on cognitive function and identify effective target images for remote social intervention.

## Abbreviations

HRQOL: health-related quality of life
KCL: Kihon checklist
MMSE: Mini-Mental State Examination
NfL: neurofilament light chain
PICMOA: Photo-Integrated Conversation Moderated by Application
PICMOA-2: Photo-Integrated Conversation Moderated by Application version 2
RCT: randomized controlled trial
UCLA-LS 10: University of California, Los Angeles Loneliness Scale-10 item version
